# Removal of Septic Infectious Matter with a View of Relieving Phlegmasia Alba Dolens*Read before Medical Association of Georgia, April, 1894.

**Published:** 1894-07

**Authors:** Geo. H. Noble

**Affiliations:** Atlanta, Ga.


### Southern Medical Record.

A MONTHLY JOURNAL OF MEDICINE AND SURGERY.

VOL. XXIV. ATLANTA, GA., JULY, 1894. NO. 7.

### Original Articles.

### REMOVAL OF SEPTIC OR INFECTIOUS MATTER WITH A VIEW OF RELIEVING PHLEGMASIA ALBA DOLENS.*

BY GEO. H. NOBLE, M.D., ATLANTA, GA.

In several instances I noticed the removal of pus-tubes was followed by marked improvement of phlegmasia alba dolens when coexistent, and believing that every case of phlegmasia has a septic origin, the conclusion was reached that toxic materials producing the disease should be removed. Carrying this idea into effect as far as practicable, results were attained that deserve some notice, and that they may arrest your attention, I make this report, together with a few remarks relative to sepsis as a causative factor.

It is a common observation that sepsis is a constant attendant of the disease, and the latter presents no symptoms that cannot reasonably be attributed to the absorption of poisonous ptomaïnes.

In the light of recent observations non-puerperal phlebitis and thrombosis are identical with that of puerperal origin. Again, phlegmasia may occur in the non-puerperal state, or the male. There must, therefore, be a common cause other than the puerperium.

Thrombosis is always preceded by phlebitis (Senn). If septic the clot will have a great tendency to extend until overcome by

*Read before Medical Association of Georgia, April, 1891.

successful phagocytosis. If not septic the inflammation will be limited, the clot small and less inclined to grow into the larger vessels.

I am inclined to believe, however, that phlebitis is always septic in origin, and, if I understand the recent teaching of Boucard, find support in this idea. The mild cases, supposed to be non-infectious, are *feebly* septic and quickly overcome by the phagocytic elements of the blood. This would prove an easy solution of the problem—the occurrence of phlegmasia under apparently different conditions.

This common cause is preceded by conditions favoring thrombosis and sepsis, such as anemia, hemorrhage, febrile conditions and consequent increase in fibrin and other factors predisposing to unsuccessful phagocytosis.

The primary lesion is usually in the genital canal, though it is confined to no particular tissue; it may occur in the bony structure, as in osteomalacia, or even in the mammary glands; an instance of which the writer saw was due to a crutch wound.

There is no specific germ. It may be putrefactive bacteria with the absorption of preformed ptomaïnes, followed by spirillæ, streptococcus pyogenes or other cocci that multiply and form poisonous ptomaïnes after penetrating the tissues, the streptococcus pyogenes being perhaps the most common, and usually associated with other vegetable ferments.

Extension to the affected limb may take place by either of two ways, or by both. Using the streptococci pyogenes as representative cocci, we trace them from the infection antrum, the clot in the ends of the uterine sinuses, through which they spread most rapidly in the axial direction of the vein until they pass beyond the confines of the clot and enter the blood in the vessels. There a battle takes place between them and the phagocytes, resulting in coagulation of the latter, temporarily checking the advance of the cocci. But the vitality of the subject being impaired or the phagocytes outnumbered, this barrier is again penetrated and the battle renewed. This is repeated from time to time with or without the chill and fever, the phagocytes gradually falling back in deference to the periodical extension of the clot until it reaches a vein of large caliber, where it may cease to grow. Here the effect of the phagocytes is evident, being sufficiently numerous and powerful to successfully destroy or carry away the invading army of germs.

If the thrombus continues to grow after reaching a large vein, it mechanically obstructs the vessel and lessens the rapidity of the blood-current, rendering the conditions more favorable for its extension. When septic, the rapidity of its growth is very much increased.

Extension to the affected limb by the other avenue, the lymphatics, is similarly progressive—that is, there are frequent attempts at arrest of the poisonous stream by coagulation successively in a number of glands in a chain. This continues until a number of lymph-ducts are obstructed and a condition developed that has been called "lymphatic œdema."

The walls of a vein at a point distant from the infection antrum may become inflamed and softened by a septic lymph-gland in close proximity. The intima is roughened, and over this surface the red corpuscles lay the foundation upon which the white corpuscles build the thrombus. This at first acts as a barrier to shut off the cocci as they penetrate the inflamed vein. If phagocytosis is successful the clot is limited and is known as an aseptic or white clot. But if the bacteria penetrate this barrier they enter the blood where coagulation is rapid upon the surface of the thrombus, and layer after layer is added until the vessel is occluded. This constitutes the septic or red clot.

Phlegmasia in a limited way occurs more frequently in the true pelvis than we are willing to admit. It may be precipitated by traumatisms surgical or otherwise, and pass unnoticed for the want of external or leg symptoms.

Recently a Philadelphia surgeon published an article in which he gives details of a number of cases of phlegmasia following celiotomy for pus-tubes, ovaries, etc., and one after a case of hysterorraphy. He stated there was nothing septic about them, no rise in temperature, and that they all made good recoveries, but he is unable to explain the cause.

The conditions favoring septic phlebitis were present in those cases of pus accumulation before the operations were done, and as it is a well known fact that the cocci penetrate the effused or coagulated lymph and softened tissues surrounding such masses, numerous colonies must remain after completing the operation, as it is often impossible to remove every vestige of the infected tissues. Thus the operation diminishes the quantity or intensity of infectious matters, but the remaining germs are often sufficient to infect a clot in the open end of a lacerated vein, which may extend to the vessels supplying the leg after the subsidence of the fever attending the operation. Under such circumstances the fever must be more or less septic, but quickly overcome by the phagocytic elements of the tissues.

Fever however, is not an essentially constant symptom of sepsis. Low temperature in purulent peritonitis and the absence of increased temperature in large pelvic abscesses occasionally demonstrate the fact.

It is not sound logic to urge the idea of a non-septic origin in these cases, because there was no continued elevation in temperature and an absence of chills.

Nor is it safe to say that the case of hysterorraphy was free from sepsis, as no man's ligature is infallible, and as it is occasionally observed that an unclean ligature may remain incased in exudate for a limited period without the slightest signs of sepsis, to eventually, suppurate and escape. If the abdominal cavity will tolerate this, why may we not have septic matter introduced in a simple hysterorrarphy resulting in fever that may go hand in hand with the fever of fermentation following the operation, and subsiding as it does, or be mistaken for it ? Again, so many cases so close together is another link in the chain of suspicion that casts a shadow upon the purity of the ligatures.

The cases that first arrested my attention regarding the improvemeut in phlegmasia alba dolens after removing septic or infectious matter from the pelvis, were three in number—two double pyosalpynx, one of which had an abscess of the left ovary of about two ounces in capacity ; the third had a pus-tube on the left side. In each case the phlegmasia was in the left leg.

Though celiotomy was done primarily for the removal of diseased organs, the beneficial effect upon the swelled leg was so apparent that it could not have escaped the most careless observer; so it occurred to me that in the future it would be desirable to remove the source of infection or septic accumulation when accessible.

I.—On the 5th day of May, 1892, I dilated and curetted the womb of Mrs. J. P. A. for hypertrophied and softened mucosa. The discharges were copious, muco-purulent and offensive, with some admixture of blood. She had been confined thirty-one days previously, chill on the third day, rapid heart action, pain in the pelvis, distention of the abdomen, cessation lochia, etc. Her physician ascribed the disturbance to "milk fever."

In the second week phlegmasia developed in the left leg. Pain intense, swelling marked, lymphatics tender, femoral vein distended and sensitive to the touch; temperature $105\frac{1}{5}°$; pulse 130; very anemic, and great loss of strength. Reduction in temperature attended the operation and an improvement was not slow to follow. The third day the limb had lost some of the hard brawny feeling, and two days later the swelling had perceptibly diminished and the soreness was greatly relieved. The improvement was steady, patient being dismissed fourteen days later.

II.—August 15, 1892, Mrs. P. E. N., phlegmasia of the left leg; was confined twenty-three days before. She gave no history of sepsis except that she claimed to have had some fever when the milk came. The disease developed in the second week. Pain severe, swelling moderate, but hard and brawny. The vaginal discharges were free but foul smelling, and consisted of pus, serum and mucus. The base of the broad ligaments thickened, cervix uteri recently lacerated upon the left side; temperature $102\frac{1}{5}°$; pulse 120, compressible; surface pale; said she had hemorrhage in her confinement. The curette brought away some placental débris and shreds of membrane. She improved rapidly and was dismissed on the sixteenth day.

III.—Mrs. G. C., admitted to the Grady Hospital June 19, 1893, a subject of septo-sapremia, following the delivery of a dead fetus. May 27th, secretions profuse, purulent and exceedingly offensive. A distended pus-tube (left) discharging through the uterus; perineum lacerated and in a state of ulceration; mucous membrane very much hypertrophied and softened; uterus fatty and very fragile; leg swelled to more than normal size. Intense pain and redness on inner side of left limb from Poupart's ligament to the foot; very pale and anemic, and slightly emaciated; temperature $102\frac{3}{5}°$; pulse 84. Was put on tonic and stimulant treatment, with phenacetine to control the fever, and antiseptic douches.

In curetting on July 5th the uterus was found very soft, especially on the left side, where a spot gave to the instrument the sensation of have penetrated a mural abscess. Great care was necessary to avoid puncturing the uterine walls. A quantity of pus and fatty débris was removed; the temperature range was from 99° to 101° for the first six days, but afterwards normal. The fever decreased with the discharges, and the patient was convalescent and dismissed free of the phlegmasia in twenty-eight days after the curetting. Perhaps removal of the diseased appendage would have been the ideal operation, but as it was, the safer course gave satisfaction.

Do not infer from the above that I advocate operative interference in every case of phlegmasia, for there are numbers that will recover if left to themselves; again it is in a limited proportion of cases only that the septic matter is accessible to the surgeon's instruments. But where a pus accumulation can be removed from the pelvis or placental débris and other septic matter can be scraped from the uterus, it is but just and right that it be done to lessen the dosage of infection and encourage successful phagocytosis.

